# The Impact of Protein Supplementation on Exercise-Induced Muscle Damage, Soreness and Fatigue Following Prolonged Walking Exercise in Vital Older Adults: A Randomized Double-Blind Placebo-Controlled Trial

**DOI:** 10.3390/nu12061806

**Published:** 2020-06-17

**Authors:** Dominique S. M. ten Haaf, Coen C. W. G. Bongers, Hugo G. Hulshof, Thijs M. H. Eijsvogels, Maria T. E. Hopman

**Affiliations:** 1 Radboud Institute for Health Sciences, Department of Physiology, Radboud University Medical Center, 6500 HB Nijmegen, The Netherlands; Dominique.tenhaaf@live.nl (D.S.M.t.H.); Coen.Bongers@radboudumc.nl (C.C.W.G.B.); hugo.hulshof@gmail.com (H.G.H.); Maria.Hopman@radboudumc.nl (M.T.E.H.); 2 Division of Human Nutrition and Health, Wageningen University, 6708 PB Wageningen, The Netherlands

**Keywords:** muscle damage, elderly, physical activity, endurance exercise, milk protein concentrate

## Abstract

Background: It is unknown whether protein supplementation can enhance recovery of exercise-induced muscle damage in older adults who have a disturbed muscle protein synthetic response. We assessed whether protein supplementation could attenuate exercise-induced muscle damage and soreness after prolonged moderate-intensity walking exercise in older adults. Methods: In a double-blind, placebo-controlled intervention study, 104 subjects (81% male, ≥65 years) used either a protein (n = 50) or placebo supplement (n = 54) during breakfast and directly after exercise. Within a walking event, study subjects walked 30/40/50 km per day on three consecutive days. Muscle soreness and fatigue were determined with a numeric rating scale, and creatine kinase (CK) concentrations and serum inflammation markers were obtained. Results: Habitual protein intake was comparable between the protein (0.92 ± 0.27 g/kg/d) and placebo group (0.97 ± 0.23 g/kg/d, *p* = 0.31). At baseline, comparable CK concentrations were found between the protein and the placebo group (110 (IQR: 84–160 U/L) and 115 (IQR: 91–186 U/L), respectively, *p* = 0.84). Prolonged walking (protein: 32 ± 9 km/d, placebo: 33 ± 6 km/d) resulted in a cumulative increase of CK in both the protein (∆283 (IQR: 182–662 U/L)) and placebo group (∆456 (IQR: 209–885 U/L)) after three days. CK elevations were not significantly different between groups (*p* = 0.43). Similarly, no differences in inflammation markers, muscle soreness and fatigue were found between groups. Conclusions: Protein supplementation does not attenuate exercise-induced muscle damage, muscle soreness or fatigue in older adults performing prolonged moderate-intensity walking exercise.

## 1. Introduction

Physical activities, i.e., resistance and endurance exercises, and especially a combination of resistance and endurance exercise with weight-bearing exercise, result in micro injuries to contractile proteins, so-called muscle damage, as demonstrated by elevated muscle soreness and an increase in plasma creatine kinase (CK) [1]. The intake of dietary proteins may augment muscle repair following acute damage through accelerating muscle protein turnover resulting in a positive net protein balance [2]. Moreover, protein consumption following muscle-damaging exercise stimulates muscle satellite cell activity [3]. Both adaptations could support the growth and repair of contractile proteins and fibers harmed during the exercise bout, via the consequent accelerated myofibrillar accretion [2,3,4]. Indeed, several studies in young males have shown beneficial effects of protein supplementation on the extent of muscle damage after high-intensity endurance exercise [5,6,7,8].

In older adults, the muscle protein synthetic response to anabolic stimuli such as protein intake is attenuated [9,10]. The anabolic resistance in older adults is multifactorial but includes a lower amino acid delivery to and uptake by ageing muscles, and an impaired mammalian target of rapamycin complex 1 (MTOR-1) pathway, which is essential for muscle protein synthesis [9,10]. Therefore, it has been suggested that older adults need higher total protein intake to counteract the attenuated post-prandial muscle protein turnover [11]. The benefits of protein supplementation for increased phosphorylation of mTOR [12,13] and on increasing muscle mass and physical performance have been extensively discussed in randomized clinical trials, systematic reviews and meta-analyses [14,15,16,17,18]. It is, however, unclear whether this can be extrapolated to diminishing muscle damage and, thus, whether improving the protein intake with supplements in vital older adults can result in a positive protein balance and increased muscle cell activity that likely stimulates myofibrillar accretion. The stimulated myofibrillar accretion might enhance the repair of muscle fibers and therefore reduce exercise-induced muscle damage after performing prolonged moderate-intensity endurance exercise. Until now, no studies have been performed investigating the effects of protein supplementation on exercise-induced muscle damage in older adults [3].

Therefore, the aim of the present study was to determine whether protein supplementation could attenuate exercise-induced muscle damage, muscle soreness and fatigue after performing prolonged moderate-intensity endurance exercise in healthy, vital, older adults. We hypothesized that protein supplementation improves recovery from endurance exercise.

## 2. Materials and Methods

### 2.1. Subjects

The present study is a subanalysis of a larger double-blind, placebo-controlled intervention study [19], aiming to assess changes in body composition, muscle strength and physical performance at baseline and after 12 weeks of protein supplementation. Vital subjects ≥65 years old with a habitual protein intake ≤1.0 g/kg/d who were registered for the 2017 Nijmegen Four Days Marches (a annual 4 d walking event for which subjects registered to walk either 30, 40 or 50 km/d [20];) were randomly allocated to either a protein- or a placebo-supplemented group. Exclusion criteria for participation in the study were diabetes mellitus (non-fasted state >11 mmol/L, allergic or sensitive to milk proteins or lactose intolerant, chronic obstructive pulmonary disease, cancer, renal insufficiency (estimated glomerular filtration rate (EGFR) <30 mL/min/1.73 m^−1^), intestinal diseases, use of statins and involved in a heavy resistance type exercise program. These inclusion criteria were used since these factors could influence the effects on the muscle parameters. With reducing the risk for confounding factors with our in- and exclusion criteria, we did include consequently a relatively healthy and vital group of older adults. All subjects gave written informed consent prior to any experimental procedures. The study conformed to the principles of the Declaration of Helsinki and was approved by a local Medical Ethical committee (Independent Review Board Nijmegen, Study-ID: NL60137.072.16). This trial was registered at Dutch trial registry (www.trialregister.nl—NTR6488).

### 2.2. Study Design

Within the event of the Nijmegen Four Days Marches, 114 subjects performed walking exercise on three consecutive days (30, 40 or 50 km/d) and were instructed to use two supplements per day. Blood samples were collected between 12 and 36 h before the onset of exercise (baseline), and immediately after the first and third walking day to assess creatine kinase (CK) concentrations. The CK concentrations after the third walking day represented the cumulative CK concentrations 48 h after the first walking day, 24 h after the second day and directly after the third walking day. Muscle soreness and fatigue were determined at baseline, and after the first and third walking day with a numeric rating scale. Additionally, exercise characteristics, body composition, training history, dietary intake and inflammation markers were determined.

### 2.3. Protein Intervention

Subjects were instructed to consume 250 mL protein supplement (Milk Protein Concentrate (MPC)) or 250 mL iso-caloric placebo drink during breakfast and were given a second supplement within 30 min after finishing the prolonged walking exercise at the research location. Moreover, in the 12 weeks prior to the prolonged walking event, both groups consumed also one supplement with breakfast and another one after exercise, or on non-exercising days, during lunch. The protein group received an additional 31 g of protein per day using the supplements (Table 1), whereas the control group received placebo supplementation with carbohydrates (FrieslandCampina Consumer Products Europe, Wageningen, the Netherlands). Protein and placebo supplements were provided in ready-to-drink non-transparent packages of 250 mL and were vanilla flavored to mask contents. Compliance was checked at the research location. Adverse events were documented. 

### 2.4. Measurements

Body composition. Height and weight (Seca 888 scale, Hamburg, Germany) were measured and used to calculate the body mass index (BMI). Furthermore, waist and hip circumferences were measured to calculate the waist–hip ratio.

Walking exercise. In the 12 weeks prior to the walking exercise event, subjects reported their weekly walking exercise (in km) in an online questionnaire. During the walking exercise event, distance (30, 40 or 50 km per day) and daily exercise duration were reported. On the first day of walking, heart rate was measured in triplicate, and averaged, every 5 km milestone with a 2-channel ECG chest band system (Polar Electro Oy, Kempele, Finland). Data were presented as mean heart rate (beats per minute, bpm) and exercise intensity, defined as a percentage of the predicted maximal heart rate (208–0.7 *age) [21].

Dietary intake. Habitual dietary intake was assessed in the week prior to the walking exercise event using a repeated 24 h recall, which is a validated method to assess the amount and distribution of protein intake [22]. Two recall days were randomized over the week prior to the walking exercise with the restriction that no participant was assigned to two identical week days or two weekend days. The dietary intake during the exercise days was not measured. The 24 h recall was performed face-to-face or by phone by trained dieticians and coded by the same dieticians into the web-based program Compl-eat, which calculated the dietary intake using the Dutch Food Composition Database of 2016 [23]. The mean of the two recorded days represented the daily dietary intake.

Muscle soreness. A validated Numeric Pain Rating Scale (NPRS; a segmented numeric version of the visual analog scale) [24] was used where subjects could mark a pain score between no pain at all (NPRS = 0) and extremely painful (NPRS = 10) for their calves, thighs and glutes, to get an idea of the subjective muscle soreness. NPRS scores of 1–5 were considered as mild pain, 6–7 as moderate pain and ≥8 as severe pain [25]. The participants filled out this online questionnaire on a computer at baseline, and within 15 min after the first and third walking day.

Fatigue. At baseline, and within 15 min after the first and third walking day, the level of fatigue was determined with a numeric rating scale ranging from 0 (no fatigue) to 10 (worst imaginable level of fatigue) using an online questionnaire. Participants completed the questionnaire on a computer at the research center.

Blood samples. Non-fasted venous blood was drawn within 15–30 min after exercise cessation from the antecubital vein, and serum and lithium heparin samples were stored at −80 °C until further analysis. Plasma CK and C-reactive protein (CRP) were measured using Siemens Dimension Vista 1500 (Siemens Healthcare Diagnostics Inc., Tarrytown, New York, USA). The reference CK value for males is <171 units per liter (U/L) and for females < 145 U/L [18]. Interleukin (IL)6 and IL10 concentrations were determined using a multiplex electroluminescence-based cytokine assay on a MESO QuickPlex SQ120 plate imager (Meso Scale Diagnostics, Rockville, Maryland, USA). CRP, IL6 and IL10 were assessed to determine the level of inflammation. Analyses were performed by trained technicians using standard operating procedures, on a single day using the same calibration and set-up to minimize variation.

### 2.5. Statistical Analysis

Statistical analyses were performed using SPSS 22.0 software (IBM SPSS Statistics for Windows, Version 22.0 IBM Corp., Armonk, NY, USA). A per-protocol analysis was used including only those subjects that finished and completed all study procedures at baseline and on the first, second and third day of the Four Days Marches. All continuous variables were visually inspected and tested for normality with the Shapiro–Wilk test. Participant characteristics were displayed as mean ± SD or median (interquartile range (IQR)) for parametric and non-parametric continuous variables, respectively. Muscle soreness and fatigue were presented as mean ± SD, despite their non-parametric nature. Categorical variables were given as number of subjects with percentages. Baseline characteristics, muscle soreness, fatigue levels, concentrations of inflammation markers and CK levels after days 1 and 3 were compared between groups using an independent-samples t test or a Mann–Whitney U test for parametric or non-parametric continuous variables, respectively. A chi-square or Fisher’s exact test was used for categorical variables. The non-parametric alternative for the repeated measures ANOVA, the Friedman test, was used to assess if there was a time effect for muscle soreness, fatigue levels, concentrations of inflammation markers and CK concentrations over the multiple days. After checking for normality of the residuals, the change of CK between baseline and day 3 was analyzed using an ANCOVA with treatment group as a fixed factor and baseline CK as a covariate. The level of significance was set at *p* < 0.05 (two-sided).

## 3. Results

### 3.1. Subjects

**Figure 1:**
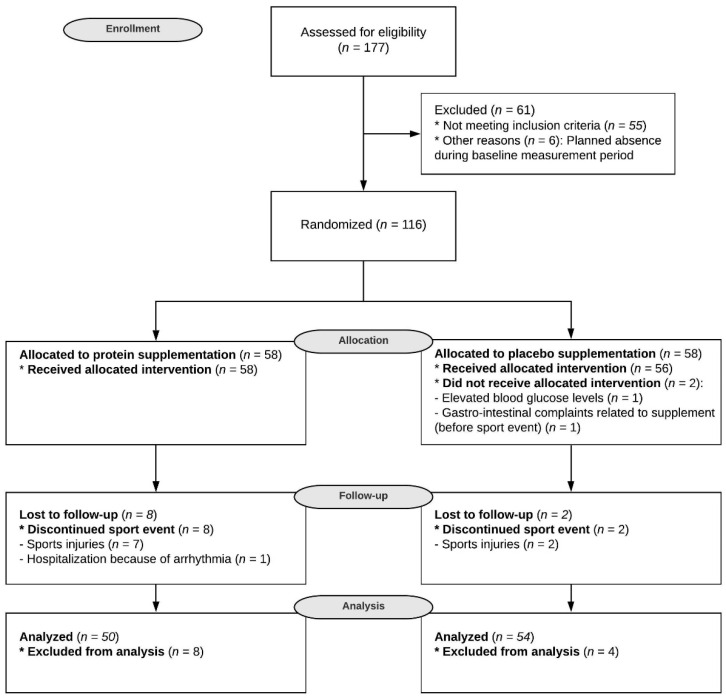
CONSORT flow diagram. This flow diagram illustrates the movement of participants through the study, which was conducted between March 2017 and July 2017. CONSORT: CONsolidated Standards Of Reporting Trials.

Before the event, two participants dropped out of the study. During the event, four subjects dropped out on the first day, two on the second day and four on the third day because they were not able to complete the walking exercise; n = 9 subjects suffered from sports injuries, and n = 1 participant reported a serious adverse event unrelated to the study (hospitalization because of arrhythmia). Although the reasons for dropouts were unrelated to the supplement, *n* = 8 subjects of the protein group dropped out compared to *n* = 2 subjects of the placebo group (Figure 1, Fisher’s exact test: *p* = 0.094).

The remaining *n* = 104 vital subjects consisted of *n* = 84 males (81%), aged 69 (IQR: 67–73) years with a BMI of 26.5 ± 2.5 kg/m^2^. Eighty participants (77%) had a BMI ≥25 kg/m^2^ and 9 participants (9%) a BMI ≥30 kg/m^2^ (obese). Both (previous) cardiovascular diseases and cancer were reported by 14 participants (14%). *N* = 50 subjects were from the protein group and *n* = 54 to the placebo group. No differences were observed between both groups for demographics and body composition (Table 2).

**Figure 2:**
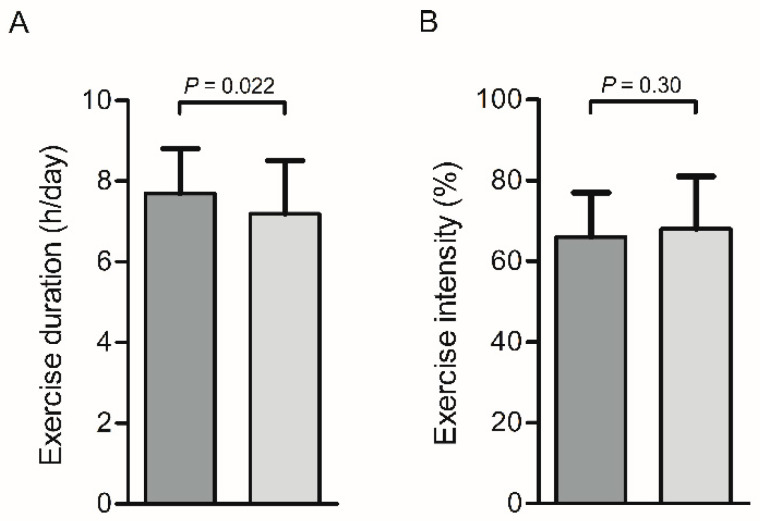
Bar charts for exercise duration (h/d) (**A**) and exercise intensity (% from HRmax) (**B**) on day 1. The exercise duration was 0.5 h longer for the protein group (dark grey) compared to the placebo group (light grey) (*p* = 0.022), whereas no significant difference between groups was observed for exercise intensity (% from maximal heartrate (HRmax) (*p* = 0.30).

The subjects’ exercise duration was 7.5 ± 1.2 h on day 1 and 7.2 ± 1.3 h on day 3. The average exercise duration of the first day was longer for the protein group (7.7 ± 1.1 h) compared to the placebo group (7.2 ± 1.3 h), *p* = 0.022 (Figure 2A). The average heart rate during walking on the first day was 104 ± 18 bpm, which equals an exercise intensity of 67 ± 12%. No differences between the protein and placebo group were observed (Table 2 and Figure 2B). Moreover, the number of trained kilometers prior to the exercise event and walking distance during the event were not different between groups (Table 2). Subjects did not perform endurance exercise activities in the three days before the event, and the subjects were not ill during the intervention.

### 3.2. Dietary Intake

Normal habitual protein intake (thus, not during the walking days) was comparable between the protein and placebo group (0.92 ± 0.27 g/kg/d and 0.97 ± 0.23 g/kg/d, respectively, *p* = 0.31) (disregarding supplements). In the protein group, *n* = 17 subjects (34%) were below the general protein recommendation of 0.8 g/kg/d, and n = 14 subjects (26%) of the placebo group were below 0.8 g/kg/d (*p* = 0.37). There were no differences between the protein and placebo group for energy intake, protein distribution, protein source and macronutrient intake (Table 2). In the 12 weeks prior to the event, the compliance was 96 ± 2% (measured with weekly online questionnaires). During the event, compliance of supplementation intake at breakfast and after finishing the walking exercise was 100%, resulting in a total protein intake of 1.28 ± 0.28 g/kg/d within the protein group.

### 3.3. Muscle Soreness and Fatigue

Muscle soreness in the calves, thighs and glutes and fatigue levels were similar between the protein and placebo groups at baseline (all *p* > 0.05, Table 3). Significant increases over time from baseline until after the third walking day were found for the calves (*p* < 0.001) and the thighs (*p* < 0.001), but not for the glutes (*p* = 0.12). Between the protein and the placebo groups, no differences were observed for muscle soreness in all muscle groups after day 1 and day 3 (all *p* > 0.05, Table 3). Similarly, categorical analysis revealed no differences between groups on all days (all *p* > 0.05, Table 4). A significant time effect from baseline until after day 3 was found for fatigue levels (*p* < 0.001), but fatigue levels were similar across groups at baseline, after day 1 and day 3 (all *p* > 0.05, Table 3).

### 3.4. Blood Analyses

**Figure 3:**
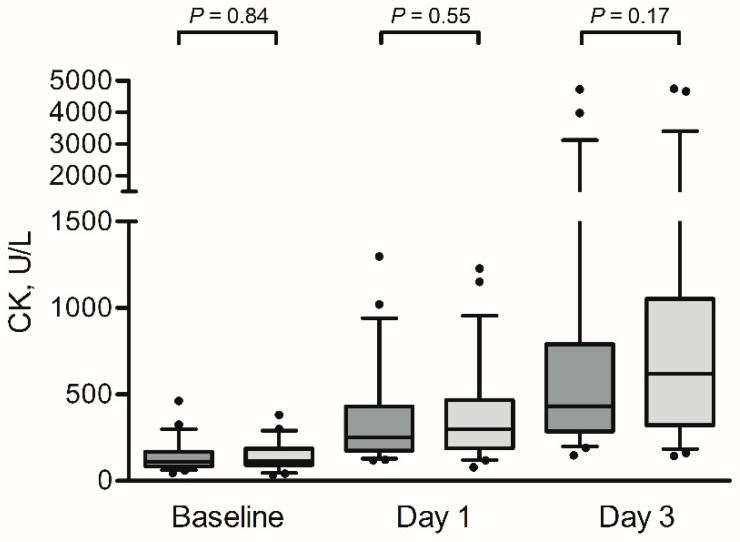
Box-and-whisker plots for creatine kinase (CK) at baseline, after day 1 and after day 3. The box-and-whisker plots represent the median, interquartile range, 5–95% percentile (upper and lower whiskers) and outliers (dots) of creatine kinase (CK) for the protein group (dark grey) and the placebo group (light grey). Prolonged walking resulted in elevated CK levels in both groups (*p* < 0.001). No significant differences were observed between the groups at the different time points (all *p* > 0.05).

At baseline, no significant differences were found in the CK concentrations between the protein and the placebo group (110 (IQR: 84–160) U/L and 115 (IQR: 91–186) U/L, respectively, *p* = 0.84). Elevated CK levels were found in the protein group after day 1 (254 (IQR: 175–445) U/L) and day 3 (433 (IQR: 288–803) U/L) and in the placebo group (day 1, 301 (188–469) U/L; and day 3, 622 (IQR: 321–1053) U/L after prolonged walking for three consecutive days (PTime < 0.001). No significant differences between groups were observed after day 1 and day 3 (Figure 3).

At baseline, *n* = 11 subjects (22%) of the protein group had CK concentrations above the reference value, which increased to *n* = 40 subjects (80%) after day 1 and n = 49 subjects (98%) after day 3. In the placebo group, *n* = 16 subjects (30%), *n* = 47 subjects (87%) and *n* = 52 (96%) had CK concentrations above the reference value at baseline, after day 1 and after day 3, respectively. The number of subjects that had CK concentrations above the reference value were not significantly different between both groups at baseline, after day 1 and after day 3 (*p* = 0.38, *p* = 0.60 and *p* = 0.53, respectively). Furthermore, the increase in CK between baseline and day 3 was not significantly different between the protein (∆283 (IQR: 182–662) U/L) and placebo group (∆456 (IQR: 209–885) U/L), *p* = 0.43.

The inflammatory markers CRP, IL6 and IL10 were similar between the protein and placebo groups at baseline (all *p* > 0.05, Table 5). Significant changes over time were seen from baseline to day 3 for CRP, IL6 and IL10 (all *p* < 0.001). No group differences were observed after day 1 (all *p* > 0.05, Table 5), but within the decreased values of IL6 and IL10 at day 3 a tendency towards lower values of IL6 and IL10 concentrations (both *p* = 0.06) was found for the protein group (Table 5).

## 4. Discussion

We were the first to assess the effect of protein supplementation on exercise-induced muscle damage after single and repetitive bouts of consecutive, prolonged, moderate-intensity exercise among vital older adults. We found a heterogeneous response in CK elevations following prolonged walking (30/40/50 km), with post-exercise CK levels of 286 (IQR: 177–461) U/L and 555 (IQR: 294–933) U/L on days 1 and 3, respectively. Protein supplementation successfully increased daily protein intake levels but did not impact exercise-induced muscle damage, as similar CK responses were found between the protein and placebo groups. Moreover, we found no differences in muscle soreness and fatigue between groups. These findings indicate that the exercise-induced muscle damage in older adults is rather high, and alternative strategies to reduce damage in the ageing muscle are warranted.

The cumulative CK following prolonged walking for three consecutive days was 555 (IQR: 294–933 U/L) in subjects aged 65 years or older, whereas reference values of <171 U/L for males and < 145 U/L for females are reported [26]. Endurance exercise significantly elevates CK levels already 3 h after exercise, but peak levels are shown approximately 72 h after exercise in eccentric muscle contraction exercise, such as walking [27]. Therefore, even higher CK levels could have occurred three days after the last exercise. Exercise-induced elevated CK levels have been proposed to be the result of leakage from muscle fibers following the mechanical tearing of the sarcolemma and opening of stretch-activated channels following contraction-induced damage [28]. While measured serum CK does not solely indicate muscle damage [29], in combination with additional measures, such as the increased muscle soreness and fatigue over time, it can assist in quantifying and substantiating muscle disturbance parameters [29]. A previous study has shown that high-intensity, short duration resistance exercise resulted in greater CK concentrations compared to low-intensity exercise for a longer duration [30]. However, the CK concentrations found after prolonged moderate-intensity walking exercise in our study are comparable with peak CK levels within 24–72 h (~480 U/L) after performing 40 min of downhill walking in young adults [31,32]. Moreover, 26 km of running- exercise resulted in peak CK levels of ~360 U/L among males with an average age of 35 years [33]. Our findings emphasize that not only short-term, vigorous-intensity activities induce muscle damage, but prolonged, weight-bearing, moderate-intensity activity performed by vital older adults leads to a similar or an even higher increase in CK.

Muscles of older adults often exhibit higher levels of damage and/or slower recovery rates compared to younger peers [28,34]. In older adults, only limited studies have been performed to determine the exercise-induced muscle damage measured by CK. After performing maximal eccentric knee extensions, peak changes in CK levels varied from ~∆20 to ~∆280 U/L within 24–48 h in adults >60 years of age [35,36]. One study assessed CK elevations after an incremental cycle ergometer test to exhaustion in older adults, but measured CK already 5 min after exercise (~∆25 U/L) [37] while peak CK levels are often found 24–48 h after exercise. Moreover, the moderate-intensity endurance activity performed by our study population was much more prolonged. This explains the greater CK elevations found in our study.

Supplemental protein has been shown to stimulate protein synthesis with concomitant reductions in protein breakdown, which is critical in remodeling damaged muscle tissue [38,39,40]. However, with increasing the protein intake from 0.97 ± 0.23 g/kg/d to 1.28 ± 0.28 g/kg/d within the protein group, which is in accordance with the recommendation of ≥1.2 g/kg/d for physically active older adults [11], we found no significant protective effect on exercise-induced CK elevations or muscle soreness. Our findings are in accordance with a study that supplemented young males with 100 g of protein immediately after 30 min of downhill running that found no differences in plasma CK concentrations and muscle soreness compared to placebo [4], but the post-exercise supplementation within that study might have been too late to prevent the exercise-induced increase in circulating CK levels. On the contrary, other studies in young males did find approximately ~200 U/L lower CK levels and lower muscle soreness at 24 h after high-intensity endurance exercise with protein supplementation compared to controls [5,6,7,8]. The supplementation strategies in these studies varied from preloading supplementation for weeks before the exercise to supplementation directly before, during exercise and post-exercise with amounts varying between 2.5 to 100 g of protein [5,6,7,8]. As our study was part of a larger study of 13 weeks, preloading of the protein intake also took place for our subjects in the 12 weeks before the walking exercise event. Moreover, 15.5 g of the total 31 g of protein of the day was used within 30 min after the exercise, in which the exercise-induced muscle protein synthetic response and enhanced preservation of skeletal muscle sensitivity to dietary amino acids was the highest [41,42,43,44]. Therefore, the supplementation strategy (amount and timing) in our study does not presumably explain the contradicting findings with earlier performed studies. Possibly, the performed exercise was insufficient to induce enough muscle damage and soreness. While CK levels do increase above the reference values as discussed earlier, the soreness only increases to maximally 1.8 out of 10. This small window could have hampered the proposed beneficial effects of protein supplementation around exercise to reduce muscle damage. We chose a pragmatic real-life study design with the benefit that you can find results that people could really experience in the field. Lab studies are, however, more controlled which could lead to more pure results and possibly larger differences. Moreover, the mentioned studies are performed in young adults [4,5,6,7,8], whereas the anabolic resistance in older adults in our study could maybe limit the efficiency of the protein supplementation. Unfortunately, only one study was found that assessed the effect of a high versus a low protein intake (three protein boluses of 3.0 versus 6.0 g/kg, respectively) within 2 h after 30 min downhill running and after a cycling time trial on exercise-induced muscle damage in (somewhat) older adults (age 52 ± 2) [44]. Although myoglobin concentrations were determined as a marker of muscle damage instead of CK, similar to our results, no statistical differences between the protein supplementation strategies were reported for muscle damage marker and muscle soreness after both trials [44]. Thus, it seems that the age-related anabolic resistance in older adults limits the possible beneficial effects of protein supplementation on exercise-induced muscle damage and soreness, for at least the first hours. It should be noted, however, that at day 3 the CK levels showed a trend to be greater in the placebo group (∆456 (IQR: 209–885) U/L) compared to the protein group (∆283 (IQR: 182–662) U/L). With the known delay in peak CK levels [27], this beneficial trend of protein supplementation might have extended in the 72 h after the last exercise.

With the increasing life expectancy across the globe, the subpopulation of older adults is growing fast. In Europe, a quarter of the population is already aged ≥60 years, and that proportion is estimated to reach 35% in 2050 [45]. To maintain the ability to remain physically active during ageing, which results in many health benefits [46], reduced exercise-induced muscle damage is favorable, especially since muscle damage is not associated with skeletal muscle hypertrophy [47], whereas the consequent muscle soreness does hinder people to exercise. Therefore, it is important to assess whether protein supplementation could reduce exercise-induced muscle damage. Unfortunately, there remains a paucity of data within older adults on this topic [3]. Our research indicates that it is harder to reduce exercise-induced muscle damage in older adults, while in younger adults often promising results are found. More studies are warranted to determine whether an even higher protein intake, possibly given during the exercise, or other strategies like creatine monohydrate and omega-3 fatty acids [3], could reduce exercise-induced muscle damage in older adults.

A limitation of the study was that we did not measure dietary intake during the exercise bouts and how many hours they slept/recovered in between the exercise bouts. Therefore, the subject’s protein intake and undertaken recovery measures, such as sleep, are unknown during these three days on which they have walked 7.0–7.5 h per day, while this could influence the muscle damage and/or soreness and, thus, our results. However, the habitual diet during the week before the walking event demonstrated similar protein intake between the protein and placebo groups. We expect, therefore, no differences in protein intake between groups during the walking exercise in our large randomized group of n = 104 subjects. Furthermore, since CK levels peak 72 h after exercise, it would be interesting to measure myoglobin levels and/or CK levels 72 days after exercise cessation. Unfortunately, we were not able to assess this within our study because this was a sub study of a larger trial, and samples were not stored in a biobank. Follow-up studies are warranted to assess dynamics of CK and myoglobin following protein supplementation. Additionally, the effects of increasing the caloric intake alone (either with protein or carbohydrate supplementation) versus a non-caloric control group were not addressed in the present study because we wanted to maintain the double-blind characteristics with regards to our subjective measurements. Future studies are, however, warranted to compare the effects of protein supplementation versus no supplementation. Finally, our group of participants was relatively healthy, and the performed exercise is atypical for habitual physical activity patterns of less vital older adults. Although the group of vital older adults is growing, more research is warranted on the effects of protein supplementation in a more heterogeneous population of older adults with different kinds of exercise that induce muscle damage.

## 5. Conclusions

In vital older adults performing prolonged moderate-intensity walking exercise, protein supplementation at breakfast and directly after exercise did not attenuate exercise-induced muscle damage, shown by large and comparable increases in CK in the protein and placebo groups after three days of consecutive walking exercise. Moreover, muscle soreness and fatigue were not affected by protein supplementation.

## Figures and Tables

**Table 1:** Nutritional composition of protein and placebo supplements.

Nutrient	Protein	Placebo
Energy, kcal/100 mL	38	39
Protein, g/100 mL	6.2	0.21
Fat, g/100 mL	0.21	1.03
Carbohydrates, g/100 mL	2.9	7.22
Of which lactose, g/100 mL	2.9	-

**Table 2:** Baseline characteristics of subjects in the protein and placebo groups.

	Total Group *n* = 104	Protein *n* = 50	Placebo *n* = 54	*p*-Value
**Demographics**				
Age, years	69 (67–73)	69 (67–72)	69 (67–73)	0.96 *
Male, n (%)	84 (81)	40 (80)	44 (82)	0.85 ^‡^
**Body composition**				
Body weight, kg	82.2 ± 10.4	83.4 ± 10.3	81.1 ± 10.4	0.27 ^§^
BMI, kg/m^2^	26.5 ± 2.5	26.7 ± 2.6	26.3 ± 2.4	0.40 ^§^
Waist–hip ratio	0.93 ± 0.07	0.93 ± 0.08	0.92 ± 0.07	0.92 ^§^
**Diet**				
Energy intake, kcal	1948 ± 514	1932 ± 505	1962 ± 527	0.77 ^§^
Protein intake, g/kg/d	0.94 ± 0.25	0.92 ± 0.27	0.97 ± 0.23	0.31 ^§^
Protein intake at breakfast, g	12.0 ± 6.1	11.0 ± 4.9	13.1 ± 6.9	0.08 ^§^
Protein intake at lunch, g	19.5 ± 10.0	18.7 ± 10.9	20.3 ± 9.2	0.42 ^§^
Protein intake at dinner, g	35.7 ± 14.5	33.3 ± 15.6	37.9 ± 13.2	0.11 ^§^
Animal protein, %	61.6 ± 10.4	61.7 ± 9.6	61.4 ± 11.3	0.88 ^§^
Plant protein, %	38.0 ± 10.6	37.6 ± 9.9	38.5 ± 11.2	0.68 ^§^
Protein, en%	16.4 ± 3.1	16.3 ± 3.3	16.5 ± 3.0	0.72 ^§^
Fat intake, en%	36.1 ± 6.7	35.5 ± 7.1	36.7 ± 6.4	0.37 ^§^
Carbohydrate intake, en%	41.9 ± 7.7	42.9 ± 7.8	40.9 ± 7.5	0.17 ^§^
**Walking exercise**				
12 week pre-event training, km	385 (257–516)	396 (288–539)	341 (244–505)	0.41 *
Distance per day				0.58 ^‡^
30 km, n (%)	72 (71)	32 (67)	40 (74)	
40 km, n (%)	25 (25)	14 (29)	11 (20)	
50 km, n (%)	5 (5)	2 (4)	3 (6)	
Mean distance per day, km	33 ± 6	32 ± 9	33 ± 6	0.60 ^§^
Heart rate (bpm)	104 ± 18	102 ± 17	106 ± 19	0.28 ^§^
Exercise intensity (% from HR_max_)	67 ± 12	66 ± 11	68 ± 13	0.30 ^§^

Data are presented as number (percentage) of subjects, mean ± SD or median (interquartile range (IQR)). ^§^ Derived by independent-samples t-test. * Derived by Mann–Whitney U test. ^‡^ Derived by chi-square test. Bpm, beats per minute; en%, energy percentage; HR_max_, predicted maximal heart rate.

**Table 3:** Muscle soreness and fatigue during moderate-intensity exercise.

	Baseline			Day 1			Day 3		
	Protein *n* = 50	Placebo *n* = 54	*p* Value	Protein *n* = 50	Placebo *n* = 54	*p* Value	Protein *n* = 50	Placebo *n* = 54	*p* Value
Calves	1.16 ± 0.47	1.15 ± 0.57	0.50	1.53 ± 0.87	1.69 ± 1.19	0.72	1.66 ± 1.36	1.64 ± 1.34	0.89
Thighs	1.10 ± 0.36	1.11 ± 0.51	0.67	1.70 ± 1.17	1.71 ± 1.54	0.51	1.83 ± 1.47	1.68 ± 1.08	0.95
Glutes	1.24 ± 0.63	1.13 ± 0.74	0.08	1.43 ± 1.15	1.37 ± 1.18	0.72	1.34 ± 0.92	1.24 ± 0.72	0.83
Fatigue	1.51 ± 1.31	1.32 ± 0.73	0.42	3.98 ± 2.06	4.15 ± 1.74	0.44	3.67 ± 1.39	3.76 ± 1.80	0.93

Data are presented as mean ± SD. *p*-values were derived from Mann–Whitney U tests.

**Table 4:** Categorical analysis of muscle soreness during moderate-intensity exercise.

	Baseline			Day 1			Day 3		
	Protein	Placebo	*p* Value	Protein	Placebo	*p* Value	Protein	Placebo	*p* Value
Calves									
No pain	44 (88)	49 (92)	0.52	31 (69)	34 (67)	1.00	33 (70)	34 (38)	0.87
Mild pain	6 (12)	4 (8)		14 (28)	16 (31)		13 (28)	15 (30)	
Moderate pain	0 (0)	0 (0)		0 (0)	1 (2)		1 (2)	1 (2)	
Severe pain	0 (0)	0 (0)		0 (0)	0 (0)		0 (0)	0 (0)	
Thighs									
No pain	46 (92)	50 (94)	0.71	31 (67)	38 (74)		29 (63)	31 (62)	0.67
Mild pain	4 (8)	3 (6)		15 (33)	11 (22)	0.26	16 (35)	19 (38)	
Moderate pain	0 (0)	0 (0)		0 (0)	2 (4)		1 (2)	0 (0)	
Severe pain	0 (0)	0 (0)		0 (0)	0 (0)		0 (0)	0 (0)	
Glutes									
No pain	43 (86)	51 (96)	0.09	36 (82)	43 (84)	0.88	40 (85)	43 (86)	1.00
Mild pain	7 (14)	2 (4)		8 (19)	7 (14)		7 (15)	7 (14)	
Moderate pain	0 (0)	0 (0)		0 (0)	1 (2)		0 (0)	0 (0)	
Severe pain	0 (0)	0 (0)		0 (0)	0 (0)		0 (0)	0 (0)	

Data are presented as n (%). *p*-values were derived from Fisher’s exact test.

**Table 5:** Blood analysis during moderate-intensity exercise.

	Baseline			Day 1			Day 3		
	Protein *n* = 50	Placebo *n* = 54	*p* Value	Protein *n* = 50	Placebo *n* = 54	*p* Value	Protein *n* = 50	Placebo *n* = 54	*p* Value
**Blood analysis**									
CRP, mg/L	2.9 (2.9–2.9)	2.9 (2.9–2.9)	0.97	2.9 (2.9–2.9)	2.9 (2.9–2.9)	0.92	9.0 (4.0–16.8)	10.0 (4.8–14.6)	0.73
IL-6, pg/mL	0.69 (0.46–0.90)	0.69 (0.43–0.97)	0.93	5.58 (3.39–11.05)	6.49 (4.65–10.93)	0.33	1.86 (1.20–2.83)	2.40 (1.41–5.46)	0.06
IL-10, pg/mL	0.20 (0.13–0.32)	0.21 (0.12–0.30)	0.87	0.33 (0.19–0.67)	0.38 (0.25–0.64)	0.35	0.19 (0.10–0.26)	0.24 (0.16–0.38)	0.06

Data are presented as median (interquartile range (IQR)). *p*-values were derived by Mann–Whitney U test. CRP, C-reactive protein; IL, Interleukin.
